# Organoleptic Chemical Markers of Serpa PDO Cheese Specificity

**DOI:** 10.3390/foods11131898

**Published:** 2022-06-27

**Authors:** Helena Araújo-Rodrigues, António P. L. Martins, Freni K. Tavaria, Maria Teresa G. Santos, Maria João Carvalho, João Dias, Nuno B. Alvarenga, Manuela E. Pintado

**Affiliations:** 1 CBQF—Centro de Biotecnologia e Química Fina, Laboratório Associado, Escola Superior de Biotecnologia, Universidade Católica Portuguesa, Rua Diogo Botelho 1327, 4169-005 Porto, Portugal; hrodrigues@ucp.pt (H.A.-R.); ftavaria@ucp.pt (F.K.T.); 2 Instituto Nacional de Investigação Agrária e Veterinária, Unidade de Tecnologia e Inovação, Avenida da República, Quinta do Marquês, 2780-157 Oeiras, Portugal; pedro.louro@iniav.pt (A.P.L.M.); nuno.alvarenga@iniav.pt (N.B.A.); 3 Geobiosciences, Geobiotechnologies and Geoengineering (GeoBioTec), Faculdade de Ciências e Tecnologias, Universidade Nova de Lisboa, 2829-516 Caparica, Portugal; joao.dias@ipbeja.pt; 4 Escola Superior Agrária, Instituto Politécnico de Beja, Rua Pedro Soares, 7800-295 Beja, Portugal; t.santos@ipbeja.pt (M.T.G.S.); joaobcarvalho@ipbeja.pt (M.J.C.)

**Keywords:** Serpa PDO cheese, chemical markers, sensory analysis, free amino acids, organic acids, volatiles

## Abstract

Serpa is a protected designation of origin cheese produced with a vegetable coagulant (*Cynara cardunculus* L.) and raw ovine milk. Despite the unique sensory profile of raw milk cheeses, numerous parameters influence their sensory properties and safety. To protect the Serpa cheese quality and contribute to unifying their distinctive features, some rheologic and physicochemical parameters of cheeses from four PDO producers, in distinct seasons and with different sensory scores, were monitored. The results suggested a high chemical diversity and variation according to the dairy, month and season, which corroborates the significant heterogeneity. However, a higher incidence of some compounds was found: a group of free amino acids (Glu, Ala, Leu, Val and Phe), lactic and acetic acids, some volatile fatty acids (e.g., iC4, iC5, C6 and C12) and esters (e.g., ethyl butanoate, decanoate and dodecanoate). Through the successive statistical analysis, 13 variables were selected as chemical markers of Serpa cheese specificity: C_3_, C_4_, iC_5_, C_12_, Tyr, Trp, Ile, 2-undecanone, ethyl isovalerate, moisture content on a fat-free basis, the nitrogen-fractions (maturation index and non-protein and total nitrogen ratio) and G’ _1 Hz_. These sensory markers’ identification will be essential to guide the selection and development of an autochthonous starter culture to improve cheese quality and safety issues and maintain some of the Serpa authenticity.

## 1. Introduction

Serpa is a Protected Designation of Origin (PDO) cheese considered one of the most famous traditional Portuguese cheeses due to its unique sensory profile and long-lasting cultural heritage regarding the manufacturing process and ingredients used [1,2,3]. This artisanal cheese is manufactured in a delimited area in the South Alentejo region from ovine raw milk and extracts of *Cynara cardunculus* L. plant pistils as a coagulant, encompassing a ripening time of at least 30 days [4,5,6,7]. 

Raw milk includes a diverse microbial community that contributes to the unique sensory attributes of raw milk cheeses. Typically, these products are characterised by a more intense aroma and flavour than cheeses produced with pasteurized milk or using other treatments [8,9]. During the Serpa cheesemaking procedure, milk pasteurisation or starter culture inoculation is not permitted, resulting in a strong flavour and semi-soft and creamy texture [1,2,4].

Despite the standardisation of the traditional production method and conditions inherent in the registration of Serpa cheese as a PDO, some physicochemical and microbiological variations in milk composition occur. Differences in manufacturing practices (often resulting from empirical knowledge) are also registered. These parameters cause product heterogeneity and strongly influence the sensory properties of raw milk cheeses and, in some cases, their safety, resulting in higher variability in the final product [10,11,12].

Regulations on PDO products state that the specific sensory characteristics and the absence of possible defects should be evaluated by the judgement of a trained and monitored panel, the “Sensory Committee”, constituted by expert judges of the specific product [13]. This panel decides whether it fulfils the minimum sensory requirements according to the expected characteristics of the product to be sold under that label. Usually, they compare the products to be analysed with the definition of the desired product. They can provide valuable descriptive information to understand better their “strong” and “weak” points [14,15].

Sensory attributes tested on different cheeses, including Serpa, to differentiate approved and unapproved products for certification comprise taste and odour or flavour, texture, the colour of the heart and rind, shape, consistency and appearance [6,15,16,17]. Besides the qualified accredited panel, a quantitative descriptive analysis (QDA) assay can also be applied: panel testing descriptive analysis is a classical methodology used to evaluate the sensory attributes of the products, which are rated by trained panellists based on a consensual vocabulary known as a lexicon [18]. 

Rejecting or assigning low scores to cheeses subject to a certified panel of tasters is often verified due to a non-consistent sensory evaluation concerning the required standard product specifications. The persistence of multiple variations in the cheese, even if small, represents a significant effect on the sensory attributes of Serpa cheese, which can determine cheese rejection and mark economic losses [19]. The microbiological and physicochemical variability may be overcome by implementing an autochthonous starter culture, improving microbiological safety and maintaining the typical aroma and flavour of the native cheese [8,10,11,20]. 

Cheese is a complex matrix, and its sensory properties result from a dynamic interaction between microbial and several biochemical parameters [8,9,10,21]. The production of numerous volatile and non-volatile metabolites by starter and non-starter microorganisms and enzymes secreted by these microorganisms and present in milk plays a crucial role in the development of the cheese organoleptic profile [8,22]. During cheese ripening, proteolysis, lipolysis and glycolysis are primary reactions from the catabolism of lipids, proteins and carbohydrates [23,24]. These reactions are followed by several biochemical mechanisms, mainly amino acids and fatty acid modifications, resulting in essential aroma and flavour-related molecules [21,22].

One of the challenges in developing a starter culture is the selection of native microbial strains that reproduce the authenticity and sensory profile connected to the original product [11,25]. In this context, Araújo-Rodrigues et al. [1] evaluate the technological and protective performance of lactic acid bacteria (LAB) isolated from Serpa PDO cheese, which constitutes an initial and essential step. However, screening flavour and texture-related compounds over time is crucial for identifying key compounds involved in the final product uniqueness [9,22].

Some free amino acids (FAAs) have a direct contribution to the typical cheese flavour and, for instance, valine (Val), leucine (Leu), isoleucine (Ile), methionine (Met), cysteine (Cys) and phenylalanine (Phe) serve as precursors to the production of numerous flavour compounds [12,26]. Short-chain fatty acids (SCFAs), such as propionic (C_3_), iso-butyric (iC_4_), butyric (C_4_) and iso-pentanoic (iC_5_), resulting mainly from FAAs’ oxidative deamination in a cheese environment, are also involved in aroma properties [12]. Other compounds, for example, medium-chain fatty acids, esters, methyl ketones, alcohols and phenolic substances, also possess a significant direct or indirect role in the final sensory profile [12,21]. However, there is a lack of scientific data on these chemical groups in Serpa cheese. 

In this study, a group of volatile compounds (free fatty acids—FFA, esters and ketones), FAAs and organic acids present in Serpa cheese with at least thirty days of ripening were investigated for the first time, analysing samples from four distinct PDO cheese producers and including four consecutive months of production, two in the winter season and two in the spring season. In addition, rheologic, physical and other chemical parameters were monitored. To better understand and protect the quality of Serpa cheese and contribute to unifying their distinctive features, an overall characterisation of cheeses with different sensory scores assigned by certified tasters was performed. 

## 2. Materials and Methods

### 2.1. Cheese Manufacture

Serpa cheeses were obtained from four PDO certified industries (identified as A, B, C and D) and manufactured according to the PDO specifications [2,6]. In each month of production, two samples of each producer were analysed from the core of ripened cheeses (at least 30 days old). The analyses were carried out during four consecutive months of production, two produced during the winter season (February and March) and two produced throughout the spring season (April and May).

### 2.2. Sensory Analysis

Sensory analysis was performed by the accredited method with the Accreditation Technical Annex L0685-1 (IPAC) with the qualified accredited panel and also through a specific developed Quantitative Descriptive Analysis for ewe cheeses similar to Serpa PDO cheeses.

#### 2.2.1. Qualified Accredited Panel

Serpa cheese has the sensory parameters (attributes and defects) and its scores well defined (Table 1), as described in the specifications book [6]. All cheeses submitted to a panel, with sensory analysis respecting the minimum total score of 14.0/20.0 in the total sensory parameters classification and a minimum score of 4.0/6.0 in the taste and smell will be accepted for PDO certification. Twelve panellists (ten females, two males, aged 28–60 years) were recruited and screened following international standards [27]. This international standard specifies criteria for the selection and procedures for the training and monitoring of selected and expert sensory assessors. Four sensory attributes were analysed: 1—cheese rind, 2—shape and consistency, 3—texture and paste colour and 4—taste and smell (Table 1).

#### 2.2.2. Quantitative Descriptive Analysis (QDA)

The descriptive sensory analysis was carried out by the panel of Serpa PDO cheese plus other selected and trained assessors. The vocabulary of sensory attributes used was developed to obtain the shortest possible list of attributes which would give a complete description of this type of ewe cheeses, but always considering the descriptors already established in the Serpa PDO cheese sensory accredited method. The attributes presented were shape, rind colour, paste colour, eyes, ammoniacal odour, grainy and buttery textures, salty, sour, spicy and bitter tastes. Each sensory attribute was evaluated on an unstructured scale from 1 to 9 points. 

The analysis was conducted in the Sensory Analysis Laboratory (LAS) of ESA/IPBEJA (Beja, Portugal). On the day of sample collection, the cheeses were brought by the certification organization in a glacier. All sessions were conducted at room temperature (18–22 °C) in a sensory panel room equipped with 8 booths with a white 6500 K lighting and a sink [28]. When at LAS, the glacier temperature was registered, then the samples coded were stored in a specific refrigerator at 3–5 °C. Prior to sensory evaluation, cheeses were held for 2 h at 18 to 22 °C. Then, they were cut into representative triangular slices (15 to 20 g) without removing the rind. Slices of cheeses were evaluated randomly in booths dedicated to sensory analysis and free from the external aroma, noise and distractions. The cheese samples were served at room temperature (18–22 °C) in a translucent plastic Petri plate coded with a random three-digit code.

The sessions were held about 3 h after breakfast. On the assessment day, the samples were presented on a common laboratory bench for visual aspect attributes and the individual samples on a Petri plate in each booth for tasting. After each sample evaluation, panellists were instructed to clean the palate with a cracker and spring water.

### 2.3. Physical Analysis

A texturometer TA.XT Plus100 texture analyser (Stable Micro Systems, Godalming, UK) was used for the instrumental texture profile analyses (TPA), at 20 ± 1 °C, adapted from Soares et al. [29]. Tests were conducted with a 100 N load cell equipped with a 20 mm Ø aluminium cylindrical probe, 20 mm of penetration depth, crosshead speed of 1.0 mms^−1^ and 5 s of delay between the first and second bite. The geometry of the tested cheeses was 3 cm in height and 16–17 cm Ø. A 0.5 cm layer was cut off from the upper surface to expose the inside for texture determinations. After, a minimum of three replicates per cheese sample were performed, evenly distributed across the surface. Finally, the force vs time texturograms were used for calculating the TPA parameter, namely: (i) hardness (N), maximum force; (ii) adhesiveness (−N.mm), the negative surface of the graph and (iii) cohesiveness (dimensionless parameter), the ratio between the work of the second bite and the work of the first bite.

Small amplitude oscillatory measurements (SAOS) of cheese samples were performed, at 20 ± 1 °C, with a controlled shear-strain rheometer (Kinexus lab+, Malvern Instruments Ltd., Malvern, UK) equipped with a 20 mm Ø serrated parallel plate geometry and 1 mm gap distance, according to Alvarenga et al. [30]. First, samples were taken from the cheese core, with an average size of around 3 cm Ø and 1.2 mm in height. After, a strain sweep test (0.01–100%) was performed at 1 Hz to identify the linear viscoelastic region (LVR). Then, samples were prepared as reported previously, using the same equipment and temperature for the frequency sweep. A steady strain of 0.01% was applied, within LVR, from 0.01 to 100 Hz, with five measurement points per decade. Storage moduli (G′_1 Hz_, in Pa) were evaluated in triplicate [31].

A colourimeter CR 300 (Minolta, Osaka, Japan) was used to perform the colour analysis and L*, a* and b* colour measurements were determined according to the CIELAB colour space, using a standard white tile (L* = 97.10, a* = −4.88, b* = 7.04) for calibration [32]. Colour measurements were repeated ten times: five measures in the core and another five in the rind of the cheese [33].

### 2.4. Chemical Analysis

#### 2.4.1. General Chemical Analysis

Titrable acidity, moisture content and total nitrogen (TN) were determined according to the Association of Official Analytical Chemistry [34] methods. In addition, pH was measured using a penetration electrode (Metrohm, Herisau, Switzerland) and fat content through the Van Gulik method [35]. Water-soluble nitrogen (WSN) was quantified by performing an aqueous extraction of the N-components, followed by nitrogen determination by the micro-Kjeldahl method using a Kjeltec System 1030 distilling coupled to a titration unit system (Tecator, Höganä, Sweden). Non-protein nitrogen (NPN) was determined by the N-component precipitation with a trichloroacetic acid (12%) solution and N determination on the filtrate (filter paper Whatman No. 42), using the micro-Kjeldahl method [36]. All chemical analyses were performed in triplicate.

#### 2.4.2. FAAs Analysis

Sample preparation for FAAs analysis was carried out according to the Pico-Tag™ method [37], with some modifications. Succinctly, for deproteinisation and FAAs extraction, 2 g of cheese were homogenised in 20 mL of perchloric acid (0.6 N), using Ultra-turrax^®^ (T18, IKA, Wilmington, DE, USA) at 12,000 rpm for 2 min. The mixture was then centrifuged at 3500 rpm for 20 min, and the supernatant was filtered with Whatman No. 1 paper. Subsequently, the pH of the filtered was adjusted (7.1 ± 0.2) and incubated on ice for 5 min. The extract was filtered using 0.45 µm filter and stored at −20 °C until HPLC analysis. During HPLC analysis, a Chromolith^®^ Performance RP18 column (4.6 × 100 mm; Merck, Darmstadt, Germany) was used. The detection was performed with a fluorimeter detector, following the method and conditions described by Pripis-Nicolau et al. [38]. For FAAs identification and quantification, 18 solutions of pure standards were prepared, and homoserine and norvaline were used as internal standards. FAAs evaluation was carried out in duplicate for each cheese sample.

#### 2.4.3. Volatile Analysis

Volatile fatty acids (VFAs), esters and ketones present in cheese samples were assessed using headspace solid-phase microextraction (HS-SPME) coupled with gas chromatography and mass spectrometry (GC–MS). For volatile analysis, 1 g of each cheese was introduced in a headspace 20 mL flask, capped with a gastight seal. Then, 10 µL of 3-octanol (internal standard; 50 mg/L) were added. For volatile adsorption, a divinylbenzene/carboxen/polydimethylsiloxane (DVR/CAR/PDMS) fibre coating (Supelco; Bellefonte, PA, USA) was used at 60 °C during 1 h. After this period, the volatiles were desorbed for 15 min in the injector port and analysed using a Varian CP-450 gas chromatograph (Walnut Creek, CA, USA) with an SGE GC column BP21 (FFAP; 50 m × 0.22 mm × 0.25 µm) from BGB Analytik (Böckten, Switzerland). The carrier gas was helium at a constant flow of 1.0 mL/min. For the mass spectra acquisition, the electron impact (EI) ionisation mode was used at 70 eV, using the temperatures in the ion source and transfer line of 210 and 160 °C, respectively. Mass spectra were scanned in the 33–350 m/z range. Data acquisition and analysis were achieved recurring to Varian Saturn 240 MS (Walnut Creek, CA, USA). Compounds identification was carried out by comparing the mass spectra of the samples with the NIST 98 MS library database. To confirm and complement the identification, mixtures of pure standards (VFAs: C_2_, C_3_, iC_4_, C_4_, iC_5_, C_5_, C_6_, C_7_, C_8_, C_9_, C_10_, C_12_; esters: ethyl acetate, butyrate, isovalerate, valerate, hexanoate, heptanoate, octanoate, nonanoate, decanoate and dodecanoate; ketones: 2-butanone, 2-heptanone, 2-octanone, 2-undecanone) were prepared and analysed under the same conditions. Thus, coupled with the mass spectra obtained, each VFA, ester and ketone retention time was also used to identify each volatile compound present in the sample. To quantify the volatiles present in cheese samples, all areas were normalized with the 3-octanol internal standard and the standard curves produced with different standard concentrations were used to calculate the concentration of VFAs, esters and ketones.

#### 2.4.4. SCFAS and Organic Acid Analysis

SCFAs and organic acids were separated and quantified by HPLC according to the conditions for sample preparation and HPLC analysis described by Sousa et al. [39], with some modifications. Briefly, 2 g of cheese were homogenised in 10 mL of sulphuric acid (13 mM) using Ultra-turrax^®^ (T18, IKA, Wilmington, DE, USA), for 2 min at 12,000 rpm. The samples were then centrifuged at 5000 rpm for 10 min. After that, the resultant supernatant was collected using a Whatman No. 1 paper. The supernatant was filtered with 0.45 µm filter and kept at −20 °C until HPLC analysis. The HPLC analysis was carried out isocratically with a cation exchange column (Aminex HPX-87H 300 × 7.8 mm column; Bio-RAD Laboratories, Hercules, CA, USA) at 0.8 mL/min and 40 °C. The mobile phase used was 13 mM of sulphuric acid. Different concentrations of pure standards were prepared and injected in the same conditions to quantify the SCFAs and organic acid concentration. Determinations were made in duplicate for each cheese sample.

### 2.5. Statistical Analysis

The panel mean scores of the quantitative descriptive data were subjected to a one-way analysis of variance (ANOVA; *p* < 0.05) and to cluster analysis to take into account an eventual panellist outlier using SPSS v. 10.0 (APSS Inc., Chicago, IL, USA). Concerning FAAs, organic acids, VFAs, esters and ketones, the SPSS statistical package 28.0 via ANOVA was used at a degree of significance of *p* < 0.05. Firstly, a normal distribution of the data was confirmed and data were compared statistically using ANOVA to understand the significance. Post-hoc multiple comparisons were carried out using Tukey’s test (α < 0.05). Principal Component Analysis (PCA) was used to identify key markers related to cheese quality and explain the main differences in the ripened cheese properties. Successive analyses were performed using STATISTICA 8.0 (StatSoft, Tulsa, OK, USA) package on different sets of chemical or biochemical markers that were sufficiently discriminative regarding cheese quality and provided greater explained total variance by the first three principal components (PC), using the most selective sensory parameters as supplementary or illustrative variables.

## 3. Results and Discussion

Traditional cheeses from raw milk harbour specific and unique sensory attributes, which are widely appreciated by consumers. These cheeses result from several technological, biochemical and microbial parameters such as temperature, time of coagulation, syneresis, water activity, pH, salt content, ripening environmental conditions as well as starter and non-starter microorganisms [8,9,30,31,40]. The study of parameters such as FAAs, volatiles, organic acids, physicochemical and rheological is essential to identify chemical markers involved in Serpa cheese uniqueness. Consequently, these related sensory compounds were screened for the first time across different production times and PDO certified producers as well as with distinct sensory classifications. 

### 3.1. Sensory Analysis

All cheeses were submitted to a sensory analysis by a specialised trained panel and Table 2 summarises the sensory results (as described in Table 1). During sensory analysis, the maximum total score was 20, with 14 being the minimum value for PDO certification and 4.0 the minimum score out of 6.0 in the taste and smell parameter. Consequently, samples with a total classification lower than 14 or with a score in the taste and smell parameter lower than 4 were classified as “bad”, between 14 and 16 as “good” and with a classification higher than 16 as “excellent”.

Generally, sensory scores of the winter season (February and March) were slightly better than in the spring season (April and May). In the winter, 2 “excellent” cheeses, 3 “good” and 3 “bad” were collected, while in the spring, 3 “excellent” and 5 “bad”. This could be explained by the lower nutritional content of pastures during high temperatures registered in the spring and summer seasons [41], typically resulting in lower microbiological quality and consequent lower sensory profile [30]. Most of the cheeses manufactured in dairy A were “excellent”, except in February, which possessed a classification of “bad”, mostly due to a low score (3.6) in taste and aroma, but in general, in both seasons, they had the higher scores in both taste and smell, as in the total score. All samples from producer B were classified as “bad” since this producer had the quality parameters below the minimum demand in both seasons.

**Figure 1:**
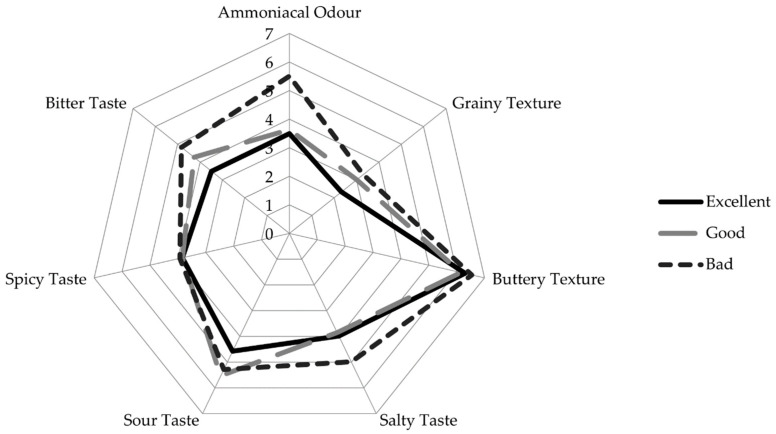
Radar graphic presenting some of the quantitative descriptive analysis (QDA) descriptors: odour (ammoniacal), texture (grainy and buttery) and taste (salty, sour, spicy and bitter) of cheese sensory characteristics. The cheeses belonging to the same sensory group (“Excellent”, “Good” or “Bad”, defined by the specialised trained panel scores) were grouped and the results were expressed as mean.

Regarding the QDA analysis, which has been applied to assess cheese sensory characteristics and their correlation with the physical–chemical properties [18], shape, rind and core colour, eyes, odour (ammoniacal), texture (grainy and buttery) and flavour (salty, sour, spicy and bitter) were evaluated. These results are summarised in Table S1. In the shape parameter, the results suggest significant differences in the “Excellent” cheeses, having this group the highest score. However, in both rind and core colour, no significant differences were revealed. Regarding ammoniacal odour, significant differences between the “Bad” cheeses (5.5) and the other categories of cheese quality were verified. In the grainy texture, the “Excellent” and “Good” groups had the lowest scores (2.31 and 3.0, respectively), with significant differences compared to the “Bad” group, showing the highest score (3.3). In the spicy and salty tastes, there were no significant differences in the three categories, but in the sour taste and bitterness, there were differences: namely, the lowest score was in the “Excellent” and the highest in the “Bad” group. Figure 1 represents the radar graphic with some of the QDA descriptors: odour (ammoniacal), texture (grainy and buttery) and taste (salty, sour, spicy and bitter), where the three classes of cheese were compared. Above all, these results in QDA taste parameters were in accordance with the scores presented by the panel in taste and aroma.

### 3.2. Physicochemical Analysis

The main physicochemical and rheological parameters of cheeses from four Serpa cheese producers were also monitored during four consecutive months of production (Table 3). The cheeses were grouped by the sensory quality, and no significant differences were verified in the physicochemical parameters evaluated except for water activity (a_w_). The results show that better quality cheeses are related to lower a_w_ values (0.90 for excellent quality and 0.92 for good quality). In previous studies, the a_w_ of Serpa PDO cheeses ranges between 0.96 and 0.98 [42,43]. The sodium chloride to moisture ratio is critical in cheese quality, as it has numerous effects, including controlling a_w_. This parameter influences cheese microorganism composition and microbial activity, mainly due to the contribution of cheese moisture and salt contents [44], which vary within traditional small dairies cheesemaking protocols [43]. These differences are likely to condition the chemical and biochemical reactions upon ripening determined by the microbiota due to selective pressure on microorganisms [45,46,47]. 

The maturation index, which corresponds to the ratio between soluble nitrogen (WSN) and total nitrogen (TN), established in the Serpa PDO cheese regulation, was set to at least 45% [6]. However, nowadays, Serpa cheese shows lower values within 30–35% [36]. The ratio WSN/TN is the only parameter regulated for Serpa PDO cheese, which means the cheese ripening evolution is based on the generic quantification of primary proteolysis. This indicator does not show significant differences concerning the cheese quality, which can be explained by the fact that it is mainly related to primary proteolysis, essentially caused by the coagulating enzymes, native milk proteinases and less related to direct or indirect microbial activity [48]. The microbial activity generally shows a more significant relationship with the quality of the cheese, mainly aroma and flavour, and can, in a way, be evaluated by indicators such as FAAs and compounds resulting from their catabolism (e.g., organic acids, FFAs and volatile compounds) or also by the ratio NPN/TN [8,9,22,45,46,47,49,50,51]. In our study, we did not find significant differences for this indicator, probably due to the great variability expressed by the high standard deviation for “bad” and “excellent” quality cheeses. However, a highest value is evident for 390 of the “good” cheeses. In a scientific study, the quotient between TN and NPN was 2.3%, TN was 3.5%, and the aminoacidic nitrogen fraction was 3.7% in Serpa cheese [33]. In PDO specifications, the moisture content on a fat-free basis (MFFB) ranges between 61 and 69% and the fat content on a dry matter (DM) between 45 and 60% [6], aligning with the results in Table 3. These values allowed the Serpa cheese classification as soft and full-fat cheese, as set by the Portuguese regulations, which generally follow the Codex Alimentarius general standard for cheese [52]. In a previous study, the protein content of Serpa was between 36 and 41 g/kg on a DM basis [7], which is in agreement with our results. Regarding pH and acidity, the previous results reported values between 4.95 and 5.7 [7,33,42,43] and 7.5 and 9.8 g of lactic acid/kg (equivalent to 8.3–10.9 mL NaOH M/100 g) in Serpa cheese [7], respectively, being in accordance with the present results. 

No significant differences were observed between the cheese groups in the evaluated rheologic parameters. The dynamic rheologic was measured through storage modulus at 1 Hz (G′_1 Hz_) and the results are presented in Table 3. Storage modulus presented values between 7868.08 and 10,589.71 Pa, similar to previous results in Serpa cheese with the same ripening time [33,36].

It should be noted that the variability obtained for the physical–chemical parameters was high, which makes statistical analysis and eventual conclusions to be drawn difficult. This fact is a recurring indication concerning traditional cheese production using raw milk, attributed to the variability of manufacturing conditions and the specificities of each cheese factory [8,9,43,47]. Accordingly, in the following sections, the cheese samples will be analysed by the dairy producer and the month of production.

### 3.3. Free Amino Acids (FAAs) Profile

Proteolysis has been extensively used to discriminate, for instance, different cheese qualities [53]. A characteristic FAA pattern is found in each cheese according to the enzymatic and amino acid inter-conversion and degradation systems. The FAAs profile depends on several cheesemaking parameters (e.g., coagulation and ripening conditions) [54]. Furthermore, the microbiota composition of raw milk and their activity directly impact cheese FAA concentration [55]. For the first time, the FAAs composition of Serpa cheeses was determined, the results being shown in Table S2. The results suggested the presence of almost all amino acids in the analysed cheeses and a quantitative variation according to the PDO producer and month of production. The results indicate significant variations according to the dairy producer in all months investigated, which corroborates the higher variability between traditional raw milk cheeses from different PDO producers. The raw milk quality and dairy environment impact the proteolytic activity of bacteria during cheese ripening [53,56]. Consequently, these facts affect the levels of FAAs according to producers and months. 

**Figure 2:**
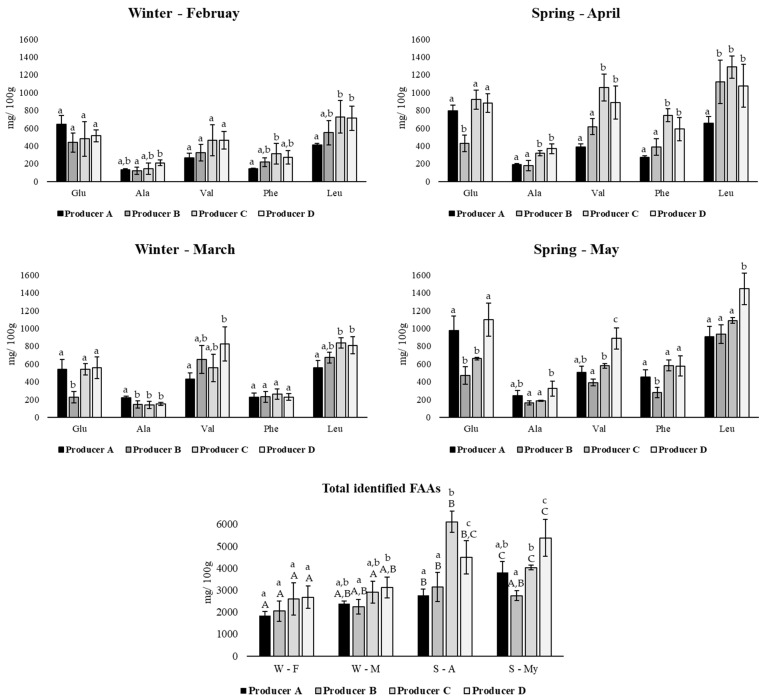
Most incident free amino acids (FAAs; mean ± standard deviation; mg/100 g) and total identified FAAs in samples from producers A, B, C and D, during four consecutive months. W—winter; S—Spring; F—February; M—March; A—April; My—May. Different superscript letters correspond to significant differences (*p* < 0.05). Lowercase letters were used to compare distinct producers (A, B, C and D) in each month (F, M, A, My), while uppercase letters were used to compare each producer during four consecutive months.

Despite the variation registered, glutamic acid (Glu), alanine (Ala), Leu, Val and Phe are the most prevalent FAAs in all samples, with concentrations ranging between 18.76 and 1445.16 mg/100 g. This group of the most incident FAAs through different months in the analysis is present in Figure 2. Generally, the following most incident FAA was aspartic acid (Asp), with concentrations varying from 42.13 to 428.49 mg/100 g. Val, Leu and Phe are essential flavour precursors [12,26]. The results may indicate that this group of FAAs is more prevalent in Serpa cheese with 30 days of ripening and probably may have an essential role in their specificity. Additionally, Serra da Estrela cheese, a traditional Portuguese PDO cheese produced with raw ewe’s milk and coagulated with *C. cardunculus* L., showed higher concentrations in some of these FAAs, namely, Leu, Phe, Ala and Val [26,57]. Concerning Terrincho cheese, a raw ewe’s milk coagulated with animal rennet, the most incident FAAs identified were Leu, Val, Asp, Glu and Pro [56]. In another study, Leu, Phe and Val are among the most prevalent FAAs in goat’s, cow’s, and cow’s mixed with goat’s milk cheeses in the final ripening time [53].

Regarding the total FAAs concentration analysed, the total concentration of FAAs identified was generally higher in the spring than in the winter (Figure 2). Significant variations in total FAAs were also verified according to the producer and month of production. In the case of producers A and D, the higher concentrations were registered in May, showing values of approximately 3805.04 and 5375.40 mg/100 g, while producers B and C, in April, had values of around 3151.56 and 6116.82 mg/100 g, respectively. Higher concentrations and accumulation of FAAs results from less conversion into volatile compounds [8].

### 3.4. Organic Acid Profile

Regarding the SCFAs profile, acetic (C_2_), propionic (C_3_) and butyric (C_4_) were identified and quantified by HPLC for the first time for Serpa cheese, with isobutyric, valeric and isovaleric by SPME-GC/MS. Additionally, lactic acid concentrations in each condition were also investigated by HPLC. Generally, organic acids are the most incident compounds in cheese [58]. Regarding HPLC analysis, the results suggest quantitative variations according to the month of production (Table 4). According to the dairy producer, significant differences were also registered. As previously said, this considerable variability may result from the differences in raw milk composition coupled with variations in the cheesemaking and ripening conditions [59]. 

Lactic acid was the most abundant acid, with concentrations between 645.03 and 2548.44 mg/100 g. Due to the metabolic processes that occur during cheesemaking and ripening, lactic acid is most abundant in similar cheeses and maturation times. In raw and pasteurised Italian cheeses manufactured with goat, sheep, cow milk or milk mixtures, lactic acid concentrations were between 199 and 3910 mg/100 g [59]. During the initial ripening phase, the residual lactose is converted into lactic acid, which plays several essential roles in metabolic reactions, such as oxidation, racemisation as well as microbial metabolism [60]. LAB are the primary lactic acid producers, which may decrease environmental pH and exert an antibacterial effect [58]. The secondary microflora is generally designated as non-starter LAB (NSLAB) and propionic bacteria, moulds and yeasts are also usually responsible for the complementary action on lactate and other cheese components derived from the primary action of the coagulant and starters (peptides, amino acids, FFAs) [23]. The antibacterial effect of lowering pH may be crucial for inhibiting foodborne pathogens’ proliferation and the action of NSLAB is important for the sensory cheese properties, mainly odour and flavour, either more directly through its metabolic activity or indirectly through the release of enzymes in the cheese matrix [61]. Organic acids are important flavour compounds and metabolites of several biochemical processes. They can have different origins, being formed from the catabolism of lactate, amino acids or fatty acids by the action of starters, secondary microflora or propionic bacteria [21,62,63]. The detection and quantification of organic acids can even be used as a classification parameter for different cheeses. The presence of these compounds may reflect the influence of the microbial metabolism [64,65].

In Serpa cheese analysed in this study, lactic acid is followed by acetic acid, the concentration ranging between 120.92 and 359.44 mg/100 g (Table 4). In Torta del Casar cheese, from sheep milk and similar to Serra da Estrela and Serpa cheeses, Ordiales et al. [45] found that acids were the most abundant volatile compounds, followed by alcohols and carbonyls. Acetic acid, a major odorant of several cheese varieties, was present in all samples analysed and in higher concentrations (15.06–45.89% of volatiles), whose origin is usually linked to residual lactose, lactate and citrate metabolism by LAB [23,61]. Garde et al. [63] found a concentration of 139.3 ± 10.7 mg/100 g in raw milk Manchego cheese, which increased to 186.4 ± 5.8 mg/100 g for the same cheese variety, showing signs of late blowing defect. The Murazzan PDO cheese is manufactured with raw sheep or a mixture of sheep and cow milk, typically within 10 days of ripening. The values of acetic acid in this Italian cheese vary between 3.8 and 124.8 mg/100 g. Concerning Robiola di Roccaverano PDO cheese, an Italian pasteurised ovine cheese inoculated with a starter culture, the acetic acid values ranged between 3.8 and 63.4 mg/100 g. Saras del Fen is also an Italian cheese manufactured with a whey mixture obtained from goat, sheep and cow milk within 20 days of ripening. In this case, the acetic acid varies from 3.3 to 75.9 mg/100 g. Acetic acid typically has higher concentrations in longer ripened cheeses, aligning with the present study results since Serpa PDO cheese possesses at least 30 days of ripening and higher values of acetic acid in the final maturation phase [59]. Greater concentrations of acetic acid and volatile organic acids such as butanoic, isobutyric and hexanoic acids are related to the sour taste of cheeses [58], and were found in Azeitão cheese, a Portuguese PDO sheep raw milk cheese made with vegetable rennet (*C. cardunculus* L.) [48].

The presence of C_3_ and C_4_ is related to the intensity of bacterial fermentation during the maturation period, typically increasing throughout this period [59]. As previously said, these acids result mainly from lactate catabolism and/or FAAs oxidative deamination in a cheese environment, being involved in aroma properties [12]. C_2_ and C_3_ have a typical pungent and vinegar odour, being major odorants of Cheddar, Emmental and Gruyère cheeses; while butyric acid has a rancid cheese-like odour, being important in the flavour of several cheese types, like Camembert, Cheddar, Grana Padano, although large amounts of this acid generally originated from the lactate butyric fermentation are undesirable [66]. C_4_ has been found widely in raw sheep milk PDO cheeses from Spain (Torta del Casar, Roncal, Manchego), Italy (CanestratoPuglise, Fiore Sardo, Pecorino Romano), Portugal (Terrincho) and also Poland (Oscypek) [48,67,68,69,70]. The C_3_ and C_4_ fermentation result from the abundant presence of secondary microorganisms and are likely to have a higher presence in old Port Salut Argentino cheese. Butyric acid concentration can be included in a group of organic acids that can significantly predict the ripening time [62].

The results suggested that C_3_ concentration ranges between 6.69 and 77.01 in the winter season and from 9.80 to 30.86 mg/100 g in the spring season. Regarding C_4_, the higher concentration was also verified in winter, with concentrations varying between 3.75 and 41.82 and in spring, between 3.13 and 31.02 mg/100 g. The results are in accordance with the literature data for similar cheeses [8,9,12,58,59]. For instance, in a study focused on Serra da Estrela cheese, the concentration of C_4_ was 44.26 mg/100 g [9]. In Torta del Casar cheese, Ordiales et al. [45] found that C_4_ and C_3_ represented 3.79–26.61% and 0.00–8.77% of volatiles, respectively, the second and third more important acids in this cheese. In goat cheese from the Muciano–Granadine breed, Buffa et al. [65] found an average of 6.1 and 8.7 mg/100 g for C_4_ and C_3_ acids, respectively, in raw milk ripened (60 days) cheese. In comparison, lower levels (2.5 and 6.9 mg/100 g, respectively) were found in pasteurised milk ripened cheese, reflecting the differences in the presence of NSLAB. 

### 3.5. Volatile Profile

Regarding the volatile composition identified for the first time for Serpa cheese, the target cheeses own a significant variability in volatile composition between producers, months and seasons of production. The results suggest the presence of several chemical groups such as volatile acids, aldehydes, aromatic compounds, ketones, alcohols and other compounds (data not shown). Numerous parameters impact volatile composition, such as raw milk quality, microbiota profile and composition (breed, feed, environmental and farming conditions) [51,71,72]. Ripening is a complex biochemical process when numerous volatiles are produced, typically increasing their concentration during this stage [73]. Vegetable coagulants improve the technological and volatile properties of cheeses. In addition, most vegetable coagulated cheeses are more digestible, resulting in a more intense flavour [51].

Despite the high variability, in the Serpa cheese samples analysed, volatile acids, esters and ketones are the most representative and typical chemical groups in other cheeses [12,58,71,73], being the target of the present study. These groups’ high variability and incidence were also found in ewe’s milk cheeses [73], Serra da Estrela PDO cheese [12], Castelo Branco PDO cheese [71] and Azeitão PDO cheese [51]. Castelo Branco is a PDO cheese, also a raw ewe’s milk cheese produced with a vegetable coagulant [74]. Native milk and microorganism lipases play important roles in cheese flavour and aroma development. In the case of raw milk, the native lipase was not deactivated by pasteurization [71]. This fact, coupled with the microbiota profile, may explain the stronger aroma and flavour of raw milk than pasteurised cheeses [51].

Despite the high variation according to the producer and month of production, the FFAs are the volatiles with higher concentrations in the winter and spring seasons (Table 5). Generally, the results suggested that the most prevalent volatile acids are iC_5_, hexanoic (C_6_), octanoic (C_8_), decanoic (C_10_) and dodecanoic (C_12_) acids. These results aligned with other studies focused on raw ewe’s milk cheeses [12,51,71,72,73]. SCFAs and medium-chain fatty acids are the main flavour contributors [71,73]. Their high content in ewe cheeses contributes to the more savoury and pungent flavour than cow cheeses [72]. In contrast, long-chain fatty acids (with more than 12 carbon atoms) have a minor impact on cheese flavour, given their relatively high perception thresholds [72,73].

C_4_ and C_6_ are two of the three most represented volatile organic compounds in Azeitão cheese, the third being 2-butanone [51]. The iC_5_ results from Leu catabolism [9] and their concentration range between 24.60 and 1400.55 mg/100 g. This macromolecule has been associated with the strong cheesy, rancid and piquant odour typically described in ripened cheeses. However, iC_5_ in higher concentrations may be unpleasant [9]. Regarding C_6_ produced by LAB through Val catabolism, it is typically correlated with soapy, goaty, waxy and sweaty aromas [9,71]. Its concentration varies from 29.69 to 861.46 mg/100 g. High concentrations of this free fatty acid were also found in Azeitão [51], Castelo Branco [71] and Serra da Estrela PDO cheese [8]. 

Concerning C_10_ and C_12_, generally, higher concentrations were registered in the winter season. These free fatty acids are associated with soapy flavours and result from bacterial enzyme activity [72]. iC_4_ and C_3_ result from starter and non-starter microbial action [9]. The concentration of iC_4_ in the winter and spring seasons ranges between 1.97 and 61.02 as well as 0.21 and 4.76 mg/100 g, respectively. This free fatty acid is derived from Leu catabolism, associated with a cheesy, rancid, sweaty and putrid aroma [9].

Esters and ketones are important volatile groups in the cheese matrix that were also monitored, present in Table 6 and Table 7. The first group can be synthesised, on the one hand, by alcohol and carboxylic acid esterification, or on the other hand, by alcohol and acyl glycerol alcoholysis reaction. Most esters found in cheese have sweet, fruity and floral notes. Some of them have a very low perception threshold, contributing to minimising sharpness and bitterness imparted from fatty acids and amines [66,75,76]. Ethyl isovalerate (0.20–5.33 mg/100 g), ethyl valerate (0.13–1.82 mg/100 g), ethyl heptanoate (0.08–8.62 mg/100 g) and ethyl nonanoate (0.19–9.34 mg/100 g) were detected in almost all cheeses analysed. Ethyl acetate (0.09–0.73 mg/100 g), ethyl butyrate (11.30–40.09 mg/100 g), ethyl hexanoate (16.9223.60 mg/100 g), ethyl octanoate (0.62–39.19 mg/100 g), ethyl decanoate (1.62–59.26 mg/100 g) and ethyl dodecanoate (5.01–12.77 mg/100 g) reached the higher average values for detected esters. Ethyl butanoate has been identified as one of the most potent odorants of Cheddar, Emmental and Pecorino. In contrast, ethyl hexanoate plays an important role in the aroma profiles of aged Cheddar, Grana Padano, Pecorino and Ragusano cheeses [66]. Ethyl octanoate was noted as significant in the aroma formation of Flor de Guia [77] and Azeitão cheeses reveal the presence of ethyl butyrate, ethyl hexanoate and ethyl octanoate [48]

Lipase activity results in ketones and FFAs release as well as flavour substances catabolism [58]. Ketones are common constituents of most dairy products. Given their typical odours and low perception threshold, they are primarily known for their contribution to the aroma of surface-mould ripened and blue-veined cheeses. In line with the other groups, some significant differences were verified according to the producer, month and season (Table 7). The ketones identified and quantified were 2-heptanone (0.52–22.91 mg/100 g), 2-octanone (0.04–1.32 mg/100 g), 2-nonanone (0.47–37.46 mg/100 g) and 2-undecanone (0.34–7.09 mg/100 g). 

Generally, despite the higher variability, the 2-heptanone and 2-nonanone were the most incident ketones identified, followed by 2-undecanone. The 2-heptanone is related to Blue cheese, Gorgonzola and Emmental cheeses aroma, while 2-nonanone is also a methyl ketone important in Gorgonzola and still in Ragusano cheese, and 2-undecanone seems to be an essential aroma in Camembert cheese. The last two methyl ketones mentioned and 2-octanone and 2-decanone are associated with fruity, floral and musty notes [66]. Ketones, though, also impact other non-mould ripened cheeses’ aroma: for instance, in Payoya goat cheese, 2-heptanone and 2-nonanone are the most important ketones [76], but they seem to be much less present in Torta del Casar cheese [42]. In Azeitão cheese, Cardinali et al. [51] identified 2-butanone, 2-pentanone and 2-heptanone.

### 3.6. Results Integration

A PCA analysis included 13 attributes (variables), previously selected as potential biochemical markers, based on the evaluation of successive analyses, trying to reduce the number of variables chosen as markers, while increasing the explained variance. The variables selected were C_3_, C_4_, tyrosine (Tyr), tryptophan (Trp), Ile, 2-undecanone, ethyl isovalerate, iC_5_, C_12_, MFFB, the nitrogen fractions (WSN/TN%, NPN/TN%) and G’_1 Hz_. The most important sensory markers, the total score for PDO Serpa cheese certification and 4 flavour attributes (spicy, bitter, ammoniacal and salty) that contribute to the cheese quality definition were used in PCA analysis as supplementary or illustrative variables to test the affinity of the selected markers with the cheese quality. The supplementary variables were projected in the variables map, although they do not influence the analysis.

**Figure 3:**
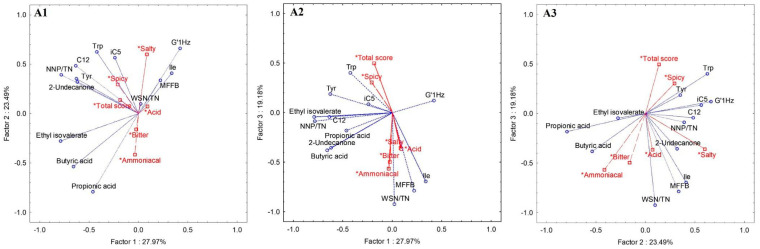
Variable projection of principal component analysis (o active variables, □ * supplementary variables): PC1 vs. PC2 (**A1**), PC1 vs. PC3 (**A2**) and PC2 vs. PC3 (**A3**). Tyr-tyrosine; Trp-tryptophane; Ile-isoleucine; iC_5_-isobutyric acid; C_12_-dodecanoic acid; NPN-non-protein nitrogen; TN-total nitrogen; WSN-water-soluble nitrogen; MFFB-moisture content on a fat-free basis.

The projection of the variables in the three planes constituted by the three principal components is shown in Figure 3. The first three principal components explained 70.6% of the total variance. As can be seen in Figure 3A1–A3, the first component (PC1) by itself condensed 28.0%, the second component (PC2) explained 23.5% of the total variance and the third component (PC3) explained 19.2% of the total variance. Therefore, the similarity map defined by the first two principal components (PC1 vs PC2) accounted for 51.5% of the total variance, PC1 vs PC3 accounted for 47.2% of the total variance and PC2 vs PC3 took into account 42.7% of the total variance. The PC1 presented negative correlations with C_4_—butyric acid (r = −0.66), Tyr (r = −0.63), 2-undecanone (r = −0.62), ethyl isovalerate (r = −0.79), C_12_ (r = −0.64) and NPN/TN% (r = −0.78) (Figure 3A1,A2); the PC2 presented negative correlations with C_3_—propionic acid (r = −0.79) and positive correlations with Trp, iC_5_ (r = 0.57) and G’_1Hz_, (r = 0.66) (Figure 3A1,A3); and the PC3 presented negative correlations with Ile (r = −0.70), MFFB (r = −0.79) and WSN/TN% (r = −0.92) (Figure 3A2,A3).

The parameter “total score” was the supplementary sensory variable that guided this study as an indicator for consideration as “Good” cheese, as obtained from the official Serpa cheese sensory panel results. The projection of the variables on the plane (Figure 3), in particular, Figure 3A2,A3, presents the sensory attribute “spicy” close to “Total score”, which indicates that such an attribute is associated with high-quality Serpa cheeses. On the other hand, sensory attributes “ammoniacal”, “bitter”, “salty” and “acid” are on the opposite side of the plane, indicating that such features are associated with lower quality cheeses when present intensely. These results are according to the sensory definition of Serpa cheese concerning taste, namely, aroma and flavour definition (Table 1)—“generally strong and with a predominance of spicy flavour”. Specifically, Serpa cheese must present a slightly spicy taste but not intense ammoniacal, bitter, acid or salty perceptions. On the other hand, the chemical parameters closer to “Total score”, representing cheeses with higher quality, are, in this order, Trp and Tyr. On the opposite side, the biochemical markers associated with poor quality cheeses are high values for WSN/TN, MFFB, Ile, butyric acid and 2-undecanone (Figure 3A1,A2—PC1 vs. PC3 and PC2 vs. PC3, respectively).

**Figure 4:**
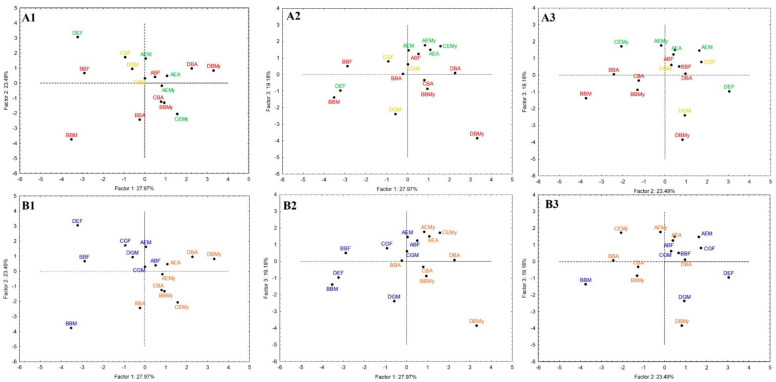
Samples projection of principal component analysis. A: highlighting samples by quality as excellent, good and bad ((**A1**): PC1 vs. PC2; (**A2**): PC1 vs. PC3 and (**A3**): PC2 vs. PC3); B: highlighting samples by season, namely, winter and spring ((**B1**): PC1 vs. PC2; (**B2**): PC1 vs. PC3 and (**B3**): PC2 vs. PC3). In the sample code, the first letter indicates the dairy (A, B, C, D), the second letter indicates the sensory quality (B-bad, G-good, E-excellent) and the last letter(s) means the month of production (F-February, M-March, A-April My-May).

The projection of the samples in the three planes constituted by the three principal components is shown in Figure 4.

From the results of the PCA analysis, it was possible to aggregate the excellent samples. Figure 4A2,A3 highlights four (out of five) samples classified as excellent (namely, AEM, AEA, AEMy and CEMy), that were found on the upper side of both planes. The concentration of “excellent” and “good” cheeses in the centre part of Figure 4A1 reflects the location of the supplementary positive sensory variable “Total score” in the plane defined by PC1 and PC2 (Figure 3A1), and the low-quality cheeses are spread over the three planes defined by the axes PC1, PC2 and PC3, suggesting the characteristic heterogeneity of the traditional cheese properties [1,2,10,12]. The excellent cheese samples were characterised, in sensory terms, by a spicy flavour and a low acid, salty, ammoniacal and bitter flavour. Likewise, these samples were characterised by high values of markers, such as Trp and Tyr, and low or intermediate values of WSN/TN, MFFB, Ile, C_4_ and 2-undecanone. On the contrary, the plan defined by PC1 and PC2 separates well, and interestingly, the cheeses according to the time of year (Figure 4B1), and this separation is maintained in Figure 4B2, not being so evident in Figure 4B3. Comparing Figure 4A1–A3 with Figure 4B1–B3, it can be concluded that even under traditional conditions, it is possible to produce “good” or “excellent” quality cheeses in either of the two periods considered.

With this study, it was possible to confirm the characteristic flavour of Serpa cheese “aroma and flavour—generally strong and with a predominance of spicy flavour”, as established in the legal regulation [6]. In addition, it was possible to select the biochemical markers that predominate at the end of ripening, resulting from proteolysis, such as the amino acids Trp and Tyr, which have been used as cheese proteolysis based ripening index [78]. Another relevant marker seems to be WSN/TN (%), which is included in the Serpa cheese certification (minimum of 45%) [6]. This is probably a regulation value to review as it is a high minimum value. As this study suggests, a more suitable minimum value should fall in the 30 and 35% range, similar to those considered for similar Portuguese cheeses, such as Serra da Estrela and Azeitão PDO cheeses.

On the other hand, PC1 could separate samples by season. From Figure 4B1,B2, it can be seen that the winter season samples predominate on the left side of the plots, with high values of butyric acid, tyrosine, 2-undecanone, ethyl isovalerate, C_12_ and NPN/TN (%) and, simultaneously, samples with low values for these markers were the spring samples, represented on the right side of the plane. This is an old widespread feeling among communities of raw milk cheese producers from different regions. Additionally, the aggregation by dairy (A, B, C and D) is also visible, which is also pointed to as a critical parameter that impacts cheese microbiota and, consequently, the final product properties [53,56].

## 4. Conclusions

Physicochemical and microbiological variations in milk composition and the differences in the manufacturing practices greatly influence the final sensory characteristics of cheese. Accordingly, the screening over time of key compounds involved in the organoleptic attributes was performed to evaluate Serpa cheeses from distinct PDO producers. This study included four consecutive months of production in the winter and spring seasons and suggested a high chemical diversity and variation according to the industry, month and season of production, which corroborates the significant heterogeneity of traditional raw milk cheeses related, for instance, to dairy technological and hygiene conditions and variation of milk composition according to the season of the year, milking and nutrition conditions [1,2].

In this study, a higher incidence of some compounds in all cheeses was found: a group of FAAs (Glu, Ala, Leu, Val and Phe), lactic and acetic acids, some VFAs (e.g., iC_4_, iC_5_, C_6_ and C_12_) and esters (for instance, ethyl butanoate, decanoate and dodecanoate). These chemical groups may play a crucial role in Serpa cheese specificity. Through successive statistical analysis, 13 variables were selected as chemical markers of Serpa cheese specificity, namely, C_3_, C_4_, Tyr, Trp, Ile, 2-undecanone, ethyl isovalerate, iC_5_, C_12_, MFFB, the nitrogen fractions (WSN/TN%, NPN/TN%) and G’_1 Hz_. This study shows the importance of proteolysis in Serpa cheese, associated with different volatile compounds, especially the negative effect of compounds linked to strong perceptions of ammoniacal, bitter, acidic and salty flavour. The high level of compounds that enhance the negative sensory attributes raises the need to reduce the presence or limit the action of the microbial groups responsible for its formation. This can be promoted by selecting microorganisms suitable for autochthonous starter culture development. In the future, the identification of these sensory markers will be important to guide the selection and development of autochthonous starter culture to improve Serpa’s quality and safety issues and, at the same time, maintain some of the Serpa authenticity.

## Figures and Tables

**Table 1:** Sensory parameters in Serpa PDO cheese certification [6].

Sensory Parameter		Scores
Cheese rind	Flat or wavy rind, thin or medium-thin; whole/continuous rind; with an intense straw yellow or lemon yellow colour and sometimes with dried moulds spots.	3.5–4.0
	Little adherent rind, malformed, difficult cheese paste containment, with slits more or less extended and open, or hard and thick, with white colour, stained or yellow-brownish intense colour.	2.0–3.0
	Deeply deteriorated, excessive thickness and deep stained.	0.0–1.5
Shape and consistency	Regular with side bulge and no sharp edges. Semi-soft consistency with some fluctuation—massive sound or slightly tympanic (when percussed by hand).	3.5–4.0
	Edges, hard consistency or excessively deformable by too much softness; sharp tympanic sound.	2.0–3.0
	Exaggerated deformation; too much fluid consistency.	0.0–1.5
Texture and paste colour	Well bonded, closed or with some eyes, and medium buttery paste; white-ivory uniform colour.	5.5–6.0
	Badly bonded, hard centres, haggard or irregular, with interstitial water; white-mate colour, white centres with irregular colouration and stains.	3.0–5.0
	Not bonded, spongy; colour white or stained with different tones.	0.0–2.5
Taste and smell	Smooth taste or slightly sharp and spicy; smooth smell or slightly strong and ammoniacal.	5.5–6.0
	Soapy, salty, bitter, strong and unpleasant, strong and sharp ammoniacal smell.	3.0–5.0
	Disgusting taste and smell.	0.0–2.5

**Table 2:** Summary of samples analysed and sensory analysis (mean ± standard deviation).

Sensory Scores								
Month	Dairy	Cheese Rind	Shape and Consistence	Texture and Paste Colour	Taste and Smell	Total	Sensory Quality	Code
W-February	A	3.6 ± 0.73 ^A,C a^	3.6 ± 0.32 ^A c^	3.7 ± 0.70 ^B b^	3.6 ± 0.44 ^B,C b^	14.5	Bad	ABF
	B	2.5 ± 0.80 ^B,C b^	2.4 ± 0.79 ^B b^	3.1 ± 0.32 ^B b^	3.3 ± 0.59 ^B b^	11.3	Bad	BBF
	C	3.4 ± 0.32 ^A a^	2.9 ± 0.42 ^B a,d^	4.5 ± 0.60 ^C a,c^	4.3 ± 0.85 ^A,C a,c^	15.1	Good	CGF
	D	3.1 ± 0.73 ^A,C a^	3.4 ± 0.35 ^A a^	5.3 ± 0.65 ^A a^	4.8 ± 0.80 ^A a^	16.6	Excellent	DEF
W-March	A	3.8 ± 0.26 ^A a^	3.7 ± 0.25 ^A a^	5.3 ± 0.50 ^A a^	5.1 ± 0.46 ^A a^	17.9	Excellent	AEM
	B	3.1 ± 0.56 ^B,b c^	3.2 ± 0.26 ^B a^	4.6 ± 0.68 ^A a^	3.1 ± 0.86 ^B a^	14.0	Bad	BBM
	C	2.8 ± 0.44 ^B b^	3.2 ± 0.36 ^B a,c^	5.1 ± 0.81 ^A a^	4.7 ± 0.94 ^A a^	15.8	Good	CGM
	D	2.9 ± 0.64 ^B a,c^	3.4 ± 0.42 ^A,B a^	4.9 ± 0.69 ^A a^	4.3 ± 0.80 ^A a^	15.5	Good	DGM
S-April	A	3.5 ± 0.50 ^A a^	3.5 ± 0.42 ^A a^	5.2 ± 0.70 ^A a^	5.0 ± 1.00 ^A a^	17.2	Excellent	AEA
	B	3.3 ± 0.27 ^A a,c^	2.8 ± 0.59 ^B a^	3.3 ± 0.44 ^B,C b^	3.1 ± 0.27 ^B b^	12.5	Bad	BBA
	C	3.3 ± 0.27 ^A a^	2.8 ± 0.65 ^A,B b,c,d^	3.8 ± 1.00 ^B b,c^	3.6 ± 0.90 ^B b,c^	13.5	Bad	CBA
	D	2.0 ± 0.47 ^B b^	2.2 ± 0.63 ^B b^	2.9 ± 0.41 ^C b^	2.6 ± 1.07 ^B b^	9.7	Bad	DBA
S-May	A	3.8 ± 0.27 ^A a^	3.5 ± 0.41 ^A a^	5.2 ± 0.39 ^A a^	4.9 ± 0.69 ^A a^	17.4	Excellent	AEMy
	B	3.4 ± 0.24 ^A a^	3.4 ± 0.24 ^A a^	3.6 ± 0.85 ^B b^	3.7 ± 0.49 ^B b^	14.1	Bad	BBMy
	C	3.4 ± 0.44 ^A a^	3.5 ± 0.60 ^A a^	5.0 ± 0.65 ^A a^	4.7 ± 0.92 ^A a^	16.6	Excellent	CEMy
	D	2.3 ± 0.39 ^B b,c^	2.0 ± 0.58 ^B b^	2.9 ± 0.19 ^B b^	3.2 ± 0.38 ^B b^	10.4	Bad	DBMy

W: winter; S: spring. Different superscript letters correspond to significant differences (*p* < 0.05). Lowercase letters were used to compare distinct producers (A, B, C and D) in each month (February, May, April, May), while uppercase letters were used to compare each producer during four consecutive months.

**Table 3:** Physicochemical and rheological parameters of cheeses grouped by sensory analysis.

Parameters		Sensory Classification		
		Bad	Good	Excellent
Physicochemical	Dry matter (%)	49.41 ± 3.12	50.97 ± 5.33	52.87 ± 3.17
	Fat (%)	27.28 ± 3.16	29.00 ± 3.54	27.73 ± 1.88
	Crude protein (%)	21.00 ± 2.02	20.95 ± 1.36	19.16 ± 2.31
	Fat content/dry matter (%)	55.23 ± 5.68	56.86 ± 0.99	52.48 ± 1.44
	MFFB (%)	69.62 ± 3.89	68.96 ± 4.08	65.25 ± 3.05
	a_w_	0.94 ± 0.01 ^a^	0.92 ± 0.01 ^b^	0.90 ± 0.01 ^b^
	Acidity (mL NaOH M/100 g)	10.19 ± 1.66	7.47 ± 0.47	10.27 ± 0.53
	pH	5.17 ± 0.23	5.09 ± 0.08	4.98 ± 0.16
	Maturation index (WSN/TN%)	40.64 ± 8.27	41.86 ± 8.06	33.81 ± 7.34
	NPN/TN (%)	5.45 ± 5.08	12.11 ± 0.89	5.45 ± 5.43
Rind colour	L*	66.85 ± 2.48	65.80 ± 3.01	65.11 ± 1.93
	a*	−1.66 ± 0.97	−2.77 ± 0.50	−3.07 ± 0.90
	b*	19.59 ± 3.01	22.40 ± 6.88	16.86 ± 4.47
Core colour	L*	80.96 ± 2.81	89.70 ± 0.74	81.64 ± 2.74
	a*	−4.07 ± 0.74	−4.03 ± 0.00	−4.48 ± 1.03
	b*	14.55 ± 2.74	14.38 ± 1.12	15.20 ± 2.39
Rheologic	G’_1 Hz_ (Pa)	7868.08 ± 4154.11	10,120.67 ± 369.11	10,589.71 ± 1545.85
	Hardness (N)	3.04 ± 2.28	3.76 ± 1.27	2.74 ± 2.10
	Cohesiveness	0.63 ± 0.11	0.68 ± 0.03	0.71 ± 0.05
	Adhesiveness (−N. mm)	16.59 ± 10.39	26.88 ± 7.52	16.36 ± 8.05

MFFB—moisture content on a fat-free basis; a_w_—water activity; Maturation index corresponds to WSN/TN; WSN—water-soluble nitrogen; TN—total nitrogen; NPN—non-protein nitrogen; G’—elastic moduli at 1 Hz. Each parameter (each line) with different superscript letters is significantly different (*p* < 0.05).

**Table 4:** Organic acids (mean ± standard deviation; mg/100 g) in samples from producers A, B, C and D, during four consecutive months of production.

Organic Acids				
Code	Lactic Acid	Acetic Acid (C_2_)	Propionic Acid (C_3_)	Butyric Acid (C_4_)
ABF	1881.26 ± 60.35 ^a A^	170.66 ± 8.71 ^a A^	11.79 ± 1.77 ^a A^	3.75 ± 1.34 ^a A,B^
BBF	992.72 ± 171.98 ^b A^	222.96 ± 22.04 ^b A^	25.98 ± 3.84 ^b A^	24.83 ± 2.50 ^b A,B^
CGF	1306.22 ± 29.35 ^a A^	173.64 ± 5.89 ^a A,B^	7.02 ± 1.23 ^a A^	5.83 ± 0.93 ^a A^
DEF	800.89 ± 97.92 ^c A^	120.92 ± 12.50 ^c A^	9.74 ± 1.81 ^a A^	21.03 ± 2.29 ^b A^
AEM	946.09 ± 187.96 ^a,b B^	318.62 ± 19.26 ^a,b B^	6.69 ± 1.76 ^a A^	5.41 ± 1.25 ^a A,B^
BBM	645.03 ± 160.41 ^a A^	359.44 ± 82.31 ^a B^	77.01 ± 16.83 ^b B^	41.82 ± 29.40 ^b A^
CGM	1700.09 ± 348.81 ^c A^	238.27 ± 41.03 ^b B^	13.21 ± 2.31 ^a B^	4.19 ± 0.56 ^a A^
DGM	1352.40 ± 383.24 ^b,c A,B^	265.05 ± 29.86 ^a,b B^	10.80 ± 3.05 ^a B^	25.07 ± 2.16 ^a,b A^
AEA	1977.70 ± 250.39 ^a A^	268.39 ± 58.89 ^a B^	11.86 ± 3.77 ^a A^	3.90 ± 0.99 ^a B^
BBA	807.88 ± 172.30 ^b A^	161.13 ± 30.87 ^b A^	30.86 ± 12.84 ^b A^	31.02 ± 6.42 ^a,b A,B^
CBA	827.18 ± 71.38 ^b B^	134.52 ± 45.73 ^b A^	22.62 ± 2.93 ^a,b C^	14.59 ± 3.76 ^c B^
DBA	2548.44 ± 374.96 ^c C^	335.32 ± 44.96 ^a C^	9.80 ± 1.29 ^a A^	4.02 ± 0.36 ^a B^
AEMy	2164.32 ± 442.68 ^a A^	226.45 ± 63.54 ^a A,B^	19.56 ± 3.37 ^a B^	3.13 ± 0.44 ^a A^
BBMy	1807.47 ± 442.04 ^a B^	171.61 ± 28.12 ^a A^	15.29 ± 4.84 ^a,b A^	6.41 ± 2.17 ^a B^
CEMy	832.37 ± 102.40 ^b B^	150.11 ± 33.35 ^a A^	21.31 ± 3.53 ^a C^	16.88 ± 3.86 ^b B^
DBMy	1475.10 ± 182.24 ^a,b B^	139.35 ± 6.45 ^a A^	10.25 ± 1.37 ^b A^	8.26 ± 2.28 ^a B^

N.D.—not detected. In the sample code, the first letter indicates the dairy (A, B, C, D), the second letter indicates the sensory quality (B—bad, G—good, E—excellent) and the last letter(s) means the month of production (F—February, M—March, A—April, My—May). Different superscript letters correspond to significant differences (*p* < 0.05). Lowercase letters were used to compare distinct producers (A, B, C and D) in each month (F, M, A, My), while uppercase letters were used to compare each producer during four consecutive months.

**Table 5:** Volatile free fatty acids (mean ± standard deviation; mg/100 g) in samples from producers A, B, C and D, during four consecutive months of production.

	Free Fatty Acids								
Code	iC_4_	iC_5_	C_5_	C_6_	C_7_	C_8_	C_9_	C_10_	C_12_
ABF	2.79 ± 1.23 ^a A^	1400.55 ± 324.99 ^a A^	9.91 ± 3.65 ^a,b^	134.36 ± 49.94 ^a A^	3.06 ± 0.42 ^a A^	59.76 ± 4.53 ^a A^	3.86 ± 0.66 ^a A^	278.00 ± 33.90 ^a A^	384.05 ± 83.98 ^a A,B^
BBF	10.01 ± 0.09 ^b A^	226.72 ± 17.75 ^b A^	98.92 ± 5.09 ^c A^	88.76 ± 21.59 ^a A^	5.04 ± 1.06 ^a,b A^	392.75 ± 101.77 ^b A^	8.78 ± 0.23 ^b A^	276.93 ± 43.20 ^a A^	828.99 ± 215.75 ^a,b A^
CGF	2.82 ± 0.98 ^a A,B^	1097.66 ± 230.07 ^a A^	5.69 ± 1.69 ^a A,B^	807.22 ± 172.90 ^b A^	6.46 ± 2.78 ^b A^	570.90 ± 83.05 ^c A^	10.56 ± 0.64 ^b A^	366.70 ± 58.66 ^a A^	701.46 ± 127.02 ^a,b A^
DEF	61.02 ± 1.53 ^c A^	1064.69 ± 372.91 ^a A^	14.42 ± 6.38 ^b A^	639.38 ± 174.68 ^b A^	6.80 ± 1.61 ^b A^	178.47 ± 62.85 ^a A^	11.39 ± 3.98 ^b A^	449.04 ± 181.58 ^a A^	1560.82 ± 891.59 ^b A^
AEM	1.97 ± 1.05 ^a A^	606.89 ± 169.32 ^a B^	5.13 ± 2.06 ^a B^	107.34 ± 34.61 ^a A,B^	5.50 ± 1.21 ^a B^	510.86 ± 111.95 ^a B^	14.21 ± 5.84 ^a B,C^	115.41 ± 38.04 ^a B^	1186.67 ± 440.45 ^a C^
BBM	11.91 ± 3.00 ^b A^	237.28 ± 45.31 ^b A^	100.62 ± 16.77 ^b A^	96.56 ± 38.31 ^a A^	5.41 ± 0.10 ^a A^	407.66 ± 158.38 ^a A^	8.88 ± 1.31 ^a A^	296.40 ± 105.73 ^b A^	891.00 ± 322.86 ^a A^
CGM	4.14 ± 0.93 ^a B^	706.37 ± 50.66 ^a B^	7.59 ± 2.51 ^a A^	861.46 ± 332.42 ^b A^	26.68 ± 10.69 ^b B^	141.47 ± 55.94 ^b B^	14.71 ± 7.05 ^a A^	115.03 ± 27.10 ^a B^	953.70 ± 238.19 ^a A^
DGM	5.13 ± 1.72 ^a B^	771.61 ± 138.14 ^a A^	4.98 ± 0.41 ^a B^	450.15 ± 117.63 ^c A^	4.94 ± 1.07 ^a A^	363.99 ± 134.43 ^a,b B^	9.02 ± 2.24 ^a A^	319.31 ± 18.4 ^b A^	1059.09 ± 78.17 ^a A^
AEA	4.76 ± 0.08 ^a B^	119.57 ± 35.21 ^a C^	5.06 ± 0.04 ^a B^	166.91 ± 45.79 ^a A^	3.15 ± 1.05 ^a A^	214.60 ± 75.37 ^a C^	6.61 ± 0.26 ^a,b B^	208.71 ± 70.48 ^a A^	712.34 ± 79.62 ^a A^
BBA	3.92 ± 0.13 ^a B^	99.55 ± 29.75 ^a B^	42.80 ± 15.31 ^b B^	413.81 ± 141.37 ^b B^	1.90 ± 0.30 ^a B^	178.38 ± 67.62 ^a B^	3.83 ± 1.47 ^b B^	178.89 ± 71.41 ^a A^	587.45 ± 78.76 ^a,b A^
CBA	2.19 ± 0.91 ^b A,C^	656.25 ± 228.82 ^b B^	3.74 ± 1.06 ^a B,C^	385.56 ± 139.96 ^a,b B^	5.28 ± 0.87 ^b A^	279.32 ± 118.65 ^a B,C^	8.29 ± 2.68 ^a A^	177.86 ± 73.38 ^a B^	332.43 ± 133.56 ^b B^
DBA	4.03 ± 0.75 ^a B^	682.57 ± 223.64 ^b A^	3.97 ± 1.39 ^a B^	442.20 ± 130.85 ^b A^	2.45 ± 0.83 ^a B^	281.06 ± 95.83 ^a A,B^	3.57 ± 1.40 ^b B^	268.61 ± 95.94 ^a A^	884.80 ± 361.05 ^a A,B^
AEMy	0.21 ± 0.08 ^a C^	156.17 ± 62.96 ^a C^	0.34 ± 0.12 ^a C^	46.73 ± 2.04 ^a,b B^	0.03 ± 0.00 ^a C^	29.81 ± 2.54 ^a A^	0.72 ± 0.12 ^a,b C^	22.50 ± 9.35 ^a,b C^	66.70 ± 8.13 ^a B^
BBMy	0.29 ± 0.05 ^a C^	52.18 ± 16.05 ^b B^	0.29 ± 0.13 ^a C^	85.74 ± 0.53 ^b,c A^	0.60 ± 0.22 ^b C^	57.00 ± 23.21 ^b B^	0.61 ± 0.20 ^b C^	25.38 ± 11.20 ^b B^	38.62 ± 2.74 ^b,c B^
CEMy	0.88 ± 0.21 ^b C^	24.60 ± 4.03 ^b C^	1.68 ± 0.15 ^a C^	29.69 ± 8.77 ^a B^	0.65 ± 0.49 ^b A^	4.30 ± 1.49 ^c C^	0.37 ± 0.11 ^c C^	3.41 ± 0.58 ^c C^	21.58 ± 13.63 ^b C^
DBMy	0.33 ± 0.21 ^a C^	76.03 ± 24.57 ^b B^	6.27 ± 2.64 ^b B^	92.27 ± 44.50 ^c B^	2.04 ± 0.19 ^c B^	14.02 ± 5.97 ^a,c C^	0.71 ± 0.25 ^a,c B^	11.17 ± 3.49 ^a,c B^	54.33 ± 14.23 ^a,c B^

N.D.—not detected. In the sample code, the first letter indicates the dairy (A, B, C, D), the second letter indicates the sensory quality (B—bad, G—good, E—excellent) and the last letter(s) means the month of production (F—February, M—March, A—April, My—May). Different superscript letters correspond to significant differences (*p* < 0.05). Lowercase letters were used to compare distinct producers (A, B, C and D) in each month (F, M, A, My), while uppercase letters were used to compare each producer during four consecutive months.

**Table 6:** Volatile esters (mean ± standard deviation; mg/100 g) in samples from producers A, B, C and D, during four consecutive months of production.

Esters										
Code	Ethyl Acetate	Ethyl Butyrate	Ethyl Isovalerate	Ethyl Valerate	Ethyl Hexanoate	Ethyl Heptanoate	Ethyl Octanoate	Ethyl Nonanoato	Ethyl Decanoate	Ethyl Dodecanoate
ABF	N.D.	N.D.	1.42 ± 0.26 ^a A^	0.40 ± 0.10 ^a A^	N.D.	0.46 ± 0.06 ^a A^	N.D.	2.36 ± 0.33 ^a A^	N.D.	N.D.
BBF	N.D.	N.D.	3.74 ± 0.56 ^b A,B^	0.98 ± 0.35 ^b A^	N.D.	1.13 ± 0.25 ^b A^	39.19 ± 11.06 ^b A^	0.91 ± 0.32 ^b A^	N.D.	N.D.
CGF	N.D.	N.D.	2.77 ± 0.98 ^b,c A^	1.17 ± 0.38 ^bA^	N.D.	N.D.	N.D.	1.88 ± 0.69 ^a A^	N.D.	N.D.
DEF	N.D.	N.D.	1.69 ± 0.72 ^a,c A^	0.33 ± 0.14 ^a A^	N.D.	0.62 ± 0.22 ^a A^	N.D.	N.D.	N.D.	N.D.
AEM	N.D.	N.D.	0.88 ± 0.30 ^a B^	0.39 ± 0.16 ^a A^	N.D.	0.72 ± 0.30 ^a A^	N.D.	0.83 ± 0.32 ^a A^	N.D.	N.D.
BBM	N.D.	N.D.	5.33 ± 2.47 ^b A^	1.82 ± 0.70 ^b B^	N.D.	1.24 ± 0.59 ^a A^	N.D.	1.17 ± 0.38 ^a A^	N.D.	N.D.
CGM	N.D.	N.D.	1.76 ± 0.08 ^a B^	N.D.	N.D.	N.D.	N.D.	0.75 ± 0.26 ^a B^	N.D.	N.D.
DGM	N.D.	33.05 ± 1.16 ^a A^	2.04 ± 0.30 ^a A^	N.D.	16.92 ± 2.29 ^a A^	1.04 ± 0.15 ^a B^	22.75 ± 8.89 ^a A^	N.D.	43.11 ± 14.88 ^a A^	10.34 ± 0.55^a A^
AEA	N.D.	N.D.	N.D.	N.D.	N.D.	0.71 ± 0.22 ^a A^	N.D.	0.52 ± 0.07 ^a A^	N.D.	N.D.
BBA	0.47 ± 0.08 ^a A^	40.09 ± 8.31 ^a A^	2.37 ± 0.39 ^a B,C^	0.61 ± 0.10 ^a A,C^	23.60 ± 6.39 ^a A^	0.49 ± 0.03 ª ^B^	N.D.	0.28 ± 0.03 ^a,c B^	N.D.	N.D.
CBA	N.D.	11.30 ± 0.93 ^b B^	1.15 ± 0.12 ^b B^	0.22 ± 0.04 ^b B^	21.38 ± 3.82 ^a A^	0.76 ± 0.17 ^a A^	27.36 ± 9.89 ^a A^	0.88 ± 0.24 ^d B^	59.86 ± 21.69 ^a A^	12.77 ± 4.40 ^a A^
DBA	0.73 ± 0.28 ^a A^	N.D.	N.D.	0.27 ± 0.11 ^b A^	N.D.	0.45 ± 0.19 ^a A^	N.D.	0.25 ± 0.09 ^c A^	N.D.	N.D.
AEMy	N.D.	N.D.	2.96 ± 0.15 ^a C^	0.50 ± 0.18 ^a A^	N.D.	8.62 ± 1.19 ^a B^	0.62 ± 0.24 ^a A^	9.34 ± 3.77 ^a B^	1.65 ± 0.40 ^a A^	5.01 ± 0.35 ^a A^
BBMy	N.D.	N.D.	0.30 ± 0.02 ^b C^	0.14 ± 0.05 ^b C^	N.D.	N.D.	N.D.	N.D.	N.D.	N.D.
CEMy	N.D.	N.D.	N.D.	0.13 ± 0.04 ^b B^	N.D.	0.25 ± 0.09 ^b B^	N.D.	0.19 ± 0.07 ^b B^	N.D.	N.D.
DBMy	0.09 ± 0.01 ^a B^	N.D.	0.20 ± 0.03 ^b B^	0.22 ± 0.02 ^b A^	N.D.	0.08 ± 0.02 ^b C^	N.D.	N.D.	N.D.	N.D.

N.D.—not detected. In the sample code, the first letter indicates the dairy (A, B, C, D), the second letter indicates the sensory quality (B—bad, G—good, E—excellent) and the last letter(s) means the month of production (F—February, M—March, A—April, My—May). Different superscript letters correspond to significant differences (*p* < 0.05). Lowercase letters were used to compare distinct producers (A, B, C and D) in each month (F, M, A, My), while uppercase letters were used to compare each producer during four consecutive months.

**Table 7:** Volatile ketones (mean ± standard deviation; mg/100 g) in samples from producers A, B, C and D, during four consecutive months of production.

		Ketones		
Code	2-Heptanone	2-Octanone	2-Nonanone	2-Undecanone
ABF	1.14 ± 0.06 ^a A^	N.D.	2.07 ± 0.13 ^a A^	0.71 ± 0.63 ^a A^
BBF	2.62 ± 0.97 ^a A^	0.43 ± 0.05 ^a A^	1.81 ± 0.00 ^a A^	3.25 ± 0.63 ^b A^
CGF	2.53 ± 0.95 ^a A,B^	0.22 ± 0.02 ^a A^	1.65 ± 0.39 ^a A^	1.21 ± 0.29 ^a A^
DEF	9.99 ± 4.01^b A^	1.32 ± 0.66 ^b A^	15.98 ± 6.87 ^b A^	7.09 ± 1.75 ^c A^
AEM	2.35 ± 0.97 ^a B^	N.D.	2.64 ± 1.12 ^a A^	0.85 ± 0.35 ^a A^
BBM	6.51 ± 0.61 ^b B^	N.D.	18.02 ± 1.81 ^b B^	1.96 ± 0.05 ^b B^
CGM	10.27 ± 2.44 ^c C^	0.42 ± 0.15 ^a B^	20.28 ± 0.08 ^b B^	1.32 ± 0.13 ^a A^
DGM	3.32 ± 0.99 ^a B^	0.63 ± 0.09 ^b B^	10.00 ± 3.28 ^c A,B^	3.69 ± 0.54 ^c B^
AEA	22.91 ± 0.34 ^a,b B^	N.D.	6.96 ± 2.48 ^a B^	0.83 ± 0.32 ^a A^
BBA	3.02 ± 1.24 ^a,b A^	N.D.	8.89 ± 1.73 ^a A^	0.66 ± 0.22 ^a,b C^
CBA	4.15 ± 0.34 ^a A^	0.35 ± 0.05 ^a A,B^	15.22 ± 5.68 ^b B^	1.19 ± 0.55 ^a A^
DBA	1.95 ± 0.52 ^b B^	0.17 ± 0.06 ^b B^	3.72 ± 1.06 ^a B^	0.19 ± 0.03 ^b C^
AEMy	3.17 ± 0.00 ^a B^	0.31 ± 0.09 ^a A^	14.90 ± 3.28 ^a C^	0.69 ± 0.07 ^a A^
BBMy	4.71 ± 1.06 ^b C^	0.40 ± 0.17 ^a A^	37.46 ± 8.18 ^b C^	4.21 ± 1.16 ^b A^
CEMy	0.81 ± 0.38 ^c B^	0.04 ± 0.02 ^b C^	0.47 ± 0.00 ^c A^	0.34 ± 0.14 ^a B^
DBMy	0.52 ± 0.04 ^c B^	N.D.	3.85 ± 0.43 ^c B^	0.54 ± 0.19 ^a C^

N.D.—not detected. In the sample code, the first letter indicates the dairy (A, B, C, D), the second letter indicates the sensory quality (B—bad, G—good, E—excellent) and the last letter(s) means the month of production (F—February, M—March, A—April, My—May). Different superscript letters correspond to significant differences (*p* < 0.05). Lowercase letters were used to compare distinct producers (A, B, C and D) in each month (F, M, A, My), while uppercase letters were used to compare each producer during four consecutive months.

## Data Availability

Data is contained within the article or supplementary material.

## Supplementary Materials

The following supporting information can be downloaded at: https://www.mdpi.com/article/10.3390/foods11131898/s1, Table S1: Sensory analysis; Table S2: Free amino acids (mean ± standard deviation; mg/100 g) in samples from producers A, B, C and D, for four consecutive months.
