# Supplementary material for: Organoleptic Chemical Markers of Serpa PDO Cheese Specificity
Source: Foods. 2022 Jun 27;11(13):1898. doi: 10.3390/foods11131898 (PMC9265577; doi:10.3390/foods11131898)
Supplement: Supplementary file 1 [file foods-11-01898-s001.zip › Table S1.pdf]

Table S1. Sensory analysis.

		Approved for certification		Not approved for certification
	Classification	Excellent	Good	Bad
Sensority Quality Scaning	Taste and Aroma	> 4.5	4 - 4.4	< 4
	Total	> 16.5	14 - 16.4	< 14
		Sensory Analysis		
Sensory evaluation for certification (according to Table 1)	Rind	3.473±0.198^{a}	3.037±0.291^{a,b}	2.827±0.577^{b}
	Shape and consistence	3.381±0.163^{a}	3.157±0.213^{a,b}	2.814±0.547^{b}
	Texture and Colour paste	5.111± 0.186^{a}	4.768±0.305^{a}	3.532±0.511^{b}
	Taste and Aroma	5.025±0.197^{a}	4.520±0.359^{b}	3.318±0.359^{c}
	Soma	16.991±0.423^{a}	15.480±0.372^{b}	12.491±1.624^{c}
Sensory analysis descriptors in QDA	Shape	6.671±0.481^{a}	5.252±1.141^{a,b}	4.612±1.712^{b}
	Rind Colour	6.197±0.749^{a}	5.757±1.209^{a}	5.635±1.148^{a}
	Paste colour	4.956±0.752^{a}	4.232±0.704^{a}	4.582±0.938^{a}
	Eyes	4.163±0.821^{b}	4.940±1.274^{b}	4.541±1.399^{a}
	Ammoniacal Odour	3.489±0.6755^{b}	3.625±0.659^{b}	5.502±0.976^{a}
	Grainy Texture	2.321±0.373^{b}	3.010±0.619^{a,b}	3.297±0.882^{a}
	Buttery Texture	6.297±0.789^{a}	6.142±0.616^{a}	6.543±1.029^{a}
	Salty Taste	3.989±1.358^{a}	3.850±1.906^{a}	4.988±1.068^{a}
	Sour Taste	4.579±0.637^{b}	5.542±0.692^{a}	5.286±0.644^{a,b}
	Spicy Taste	3.842±0.527^{a}	3.837±0.282^{a}	3.924±0.312^{a}
	Bitter Taste	3.488±0.492^{b}	4.270±0.386^{a}	4.831±0.511^{a}
