# Supplementary material for: Organoleptic Chemical Markers of Serpa PDO Cheese Specificity
Source: Foods. 2022 Jun 27;11(13):1898. doi: 10.3390/foods11131898 (PMC9265577; doi:10.3390/foods11131898)
Supplement: Supplementary file 1 [file foods-11-01898-s001.zip › Table S2.pdf]

Table S2: Free amino acids (mean ± standard deviation; mg/100 g) in samples from producers A, B, C and D, for four consecutive months.

Code	Asp	Glu	Cys	Asn	Ser	His	Gln	The	Arg	Ala	Tyr	Val	Met	Trp	Phe	Ile	Leu
**ABF**	86.41±14.16$^{a\ A,B}$	644.32±94.94$^{a\ A,B}$	0.77±0.13$^{a\ A}$	5.35±1.54$^{a\ A}$	4.69±0.94$^{a\ A}$	N.D.	15.54±2.55$^{a\ A}$	45.12±5.62$^{a\ A}$	32.31±4.85$^{a\ A}$	130.96±10.66$^{a,b\ A}$	3.72±0.32$^{a\ A}$	263.78±53.00$^{a\ A}$	19.71±1.75$^{a\ A}$	6.22±0.12$^{a\ A}$	142.56±8.75$^{a\ A}$	25.29±2.21$^{a\ A}$	411.81±18.07$^{a\ A}$
**BBF**	59.20±12.86$^{a\ A}$	438.71±107.38$^{a\ A}$	0.48±0.10$^{b\ A}$	4.14±1.45$^{a\ A}$	7.74±2.96$^{a\ A}$	1.64±0.32^{a}	12.61±3.60$^{a\ A}$	40.46±10.77$^{a\ A}$	30.41±4.48$^{a\ A,B}$	119.39±42.34$^{a\ A}$	64.81±22.44$^{b\ A}$	323.25±92.75$^{a\ A}$	46.17±12.91$^{a,b\ A}$	13.77±3.25$^{a,b\ A}$	218.29±49.46$^{a,b\ A}$	113.38±44.60$^{b\ A,B}$	547.79±135.81$^{a,b\ A}$
**CGF**	86.44±31.19$^{a\ A}$	478.22±192.70$^{a\ A}$	0.61±0.13$^{a,b\ A}$	6.15±5.22$^{a\ A,B}$	21.61±11.21$^{a\ A}$	4.24±3.96$^{a\ A}$	17.40±8.25$^{a\ A}$	70.83±30.92$^{a\ A}$	49.40±11.67$^{b\ A}$	144.44±64.46$^{a,b\ A}$	25.04±12.85$^{a,b\ A}$	462.89±174.60$^{a\ A}$	60.38±36.14$^{a,b\ A}$	13.45±2.25$^{a,b\ A}$	309.92±115.86$^{b\ A}$	123.75±44.63$^{b\ A,B}$	726.25±182.01$^{b\ A}$
**DEF**	78.22±31.35$^{a\ A}$	512.98±65.61$^{a\ A}$	0.46±0.15$^{b\ A}$	3.22±0.40$^{a\ A}$	18.07±12.14$^{a\ A,B}$	4.33±2.35$^{a\ A}$	22.05±10.80$^{a\ A}$	43.75±13.65$^{a\ A}$	28.11±5.56$^{a\ A}$	208.77±33.30$^{b\ A}$	57.15±38.51$^{b\ A}$	465.48±98.94$^{a\ A}$	78.70±24.09$^{b\ A}$	17.69±8.48$^{b\ A}$	270.66±75.23$^{a,b\ A}$	160.23±46.64$^{b\ A}$	711.30±135.74$^{b\ A}$
**AEM**	42.13±10.97$^{a\ C}$	540.67±109.82$^{a\ A}$	1.11±0.15$^{a\ A,B}$	5.13±1.07$^{a\ A}$	8.73±3.49$^{a\ A,B}$	20.00±3.94$^{a\ A}$	18.24±5.08$^{a\ A}$	55.69±7.96$^{a\ A}$	18.76±5.26$^{a,b\ A}$	217.56±18.40$^{a\ B}$	25.75±10.21$^{a\ A}$	428.85±68.08$^{a\ B,C}$	115.95±34.40$^{a\ B}$	9.72±1.67$^{a\ B}$	223.32±50.51$^{a\ A,B}$	102.15±8.89$^{a\ B}$	560.05±75.86$^{a\ A,B}$
**BBM**	76.51±24.33$^{a\ A,B}$	226.50±63.98$^{b\ B}$	2.27±0.32$^{b,c\ B}$	5.53±1.28$^{a\ A}$	6.02±2.01$^{a\ A}$	44.31±11.63$^{b\ A}$	20.57±6.67$^{a\ A,B}$	25.57±5.64$^{b\ A}$	15.28±3.75$^{b\ A}$	142.77±40.46$^{b\ A}$	7.45±1.13$^{b\ B}$	649.85±15.07$^{a,b\ B}$	57.23±13.97$^{b\ A}$	N.D.	229.14±58.50$^{a\ A}$	70.69±9.88$^{a,b\ A,B}$	671.11±58.81$^{a,b\ A,B}$
**CGM**	223.59±30.19$^{b\ B}$	537.80±68.41$^{a\ A}$	1.52±0.36$^{a,b\ B}$	3.09±0.79$^{b\ A}$	15.00±2.41$^{b\ A}$	45.36±8.09$^{b\ B}$	37.97±4.32$^{b\ A}$	122.29±22.21$^{c\ B}$	11.62±3.78$^{b\ C}$	138.40±25.72$^{b\ A}$	10.35±1.48$^{b\ B}$	556.32±89.27$^{a,b\ A}$	38.08±8.99$^{b\ A}$	N.D.	258.59±62.70$^{a\ A}$	66.00±12.00$^{b\ A}$	834.82±177.99$^{b\ A,B}$
**DGM**	93.19±28.82$^{a\ A}$	556.71±123.79$^{a\ A}$	2.54±0.69$^{c\ B}$	2.53±0.44$^{b\ A}$	4.84±1.58$^{a\ A}$	77.66±6.17$^{c\ B}$	42.61±9.37$^{b\ A,B}$	43.44±14.98$^{a,b\ A}$	24.40±3.41$^{a\ A}$	148.44±17.62$^{b\ A}$	3.28±0.37$^{b\ B}$	824.43±192.44$^{b\ B}$	109.40±19.83$^{a\ A}$	N.D.	228.08±35.94$^{a\ A}$	152.44±25.63$^{c\ A}$	809.64±94.95$^{b\ A,B}$
**AEA**	104.52±15.26$^{a\ A}$	791.98±64.30$^{a\ B,C}$	1.56±0.20$^{a\ B}$	22.47±1.42$^{a\ B}$	16.52±4.61$^{a,b\ B}$	7.78±1.94$^{a\ B}$	19.56±7.15$^{a\ A}$	94.62±20.45$^{a\ B}$	54.56±10.10$^{a\ B}$	187.83±13.54$^{a\ A,B}$	20.02±4.94$^{a\ A}$	386.20±34.08$^{a\ B}$	59.25±6.72$^{a\ C}$	5.64±1.31$^{a\ A}$	269.29±21.74$^{a\ B}$	79.22±1.5.54$^{a\ C}$	652.17±75.96$^{a\ B}$
**BBA**	89.73±18.81$^{a\ A,B}$	427.81±93.91$^{b\ A}$	0.33±0.11$^{b\ A}$	21.81±6.40$^{a\ B}$	12.83±6.92$^{b\ A,B}$	11.02±3.64$^{a\ B}$	29.09±10.72$^{a,b\ B}$	29.09±10.72$^{b\ A}$	24.83±6.72$^{b\ A,B}$	176.64±58.40$^{a\ A}$	24.27±5.87$^{a\ B}$	615.17±90.73$^{a\ B}$	58.24±18.53$^{a\ A}$	7.10±2.19$^{a\ A}$	385.87±93.81$^{a\ B}$	117.77±30.54$^{a,b\ A}$	119.41±244.79$^{b\ C}$
**CBA**	428.49±44.98$^{b\ C}$	919.62±108.94$^{a\ B}$	1.16±0.38$^{a\ A,B}$	9.81±1.73$^{b\ B}$	26.76±6.91$^{a\ A}$	10.16±4.13$^{a\ A}$	550.81±25.48$^{c\ B}$	351.32±28.76$^{c\ C}$	32.74±12.22$^{a,b\ A,B}$	316.15±27.92$^{b\ B}$	30.80±9.19$^{a\ A,B}$	1054.35±152.18$^{b\ B}$	147.90±15.97$^{b\ B}$	29.06±1.55$^{b\ B}$	743.39±74.04$^{b\ B}$	178.63±29.82$^{c\ B}$	1285.67±123.78$^{b\ C}$
**DBA**	96.86±25.22$^{a\ A}$	881.41±103.94$^{a\ B}$	3.46±0.36$^{c\ B}$	8.09±4.06$^{b\ B}$	16.40±3.79$^{a,b\ A,B}$	46.60±7.53$^{b\ C}$	63.73±21.16$^{b\ B}$	85.25±5.99$^{a\ B}$	81.80±12.25$^{c\ B}$	367.47±56.30$^{b\ B}$	3.11±0.99$^{b\ B}$	889.43±186.83$^{b\ B}$	121.88±35.58$^{b\ A}$	N.D.	588.00±128.88$^{b\ B}$	162.31±52.51$^{b\ A}$	1076.04±241.12$^{b\ B}$
**AEMy**	71.08±11.96$^{a\ B}$	974.76±164.62$^{a\ C}$	1.641±0.65$^{a\ B}$	6.01±2.24$^{a\ A}$	40.13±7.16$^{a\ C}$	14.59±1.99$^{a\ C}$	44.96±11.79$^{a\ B}$	133.25±18.83$^{a\ C}$	74.86±14.33$^{a\ C}$	243.54±57.02$^{a,b\ B}$	212.70±47.80$^{a\ B}$	506.80±67.18$^{a,b\ C}$	37.71±11.25$^{a\ A,C}$	11.83±2.22$^{a\ B}$	455.10±78.32$^{a\ C}$	69.84±10.43$^{a\ C}$	906.13±114.82$^{a\ C}$
**BBMy**	113.79±25.86$^{b\ B}$	471.70±164.62$^{b\ A}$	0.55±0.12$^{a,b\ A}$	7.98±3.06$^{a\ A}$	17.68±4.34$^{b\ B}$	7.01±1.12$^{b\ B}$	22.59±4.27$^{a\ A,B}$	137.84±48.34$^{a\ B}$	38.69±12.54$^{b\ B}$	161.12±22.08$^{b\ A}$	67.93±10.27$^{a\ A}$	391.50±39.99$^{a\ A}$	32.35±11.09$^{a\ A}$	7.09±5.13$^{a\ A}$	277.12±59.62$^{b\ A,B}$	55.58±10.96$^{a\ B}$	936.09±104.18$^{a\ B,C}$
**CEMy**	94.13±6.19$^{a,b\ A}$	659.98±15.43$^{b\ A}$	N.D.	51.89±1.06$^{b\ C}$	14.33±3.65$^{b\ A}$	N.D.	444.17±18.61$^{b\ C}$	127.06±3.14$^{a\ B}$	23.40±1.90$^{b\ B,C}$	187.05±6.66$^{b\ A}$	11.73±4.53$^{b\ B}$	578.45±27.35$^{b\ A}$	34.56±1.74$^{a\ A}$	20.60±2.66$^{b\ C}$	583.43±58.65$^{a\ B}$	111.81±9.91$^{a\ A}$	1087.15±31.73$^{a\ B,C}$
**DBMy**	247.67±11.74$^{c\ B}$	1098.24±184.63$^{a\ B}$	3.09±1.05$^{c\ B}$	8.65±1.37$^{a\ B}$	19.77±2.29$^{b\ B}$	15.15±1.97$^{a\ D}$	191.15±24.94$^{c\ C}$	91,41±19.02$^{a\ B}$	36.44±13.37$^{b\ A}$	323.28±85.28$^{a\ B}$	10.57±0.82$^{b\ B}$	888.54±119.15$^{c\ B}$	107.91±39.31$^{b\ A}$	N.D.	577.11±115.43$^{a\ B}$	311.26±108.40$^{b\ B}$	1445.16±177.18$^{b\ C}$

N.D. not detected. In the sample code, the first letter indicates the dairy (A, B, C, D), the second letter indicates the sensory quality (B – bad, G – good, E – excellent) and the last letter(s) means the month of production (F – February, M – March, A – April My – May). Different superscript letters correspond to significant differences (p < 0.05). Lowercase letters were used to compare distinct producers (A, B, C and D) in each month (F, M, A and My), while uppercase letters were used to compare each producer during four consecutive months.
